# 

**Published:** 1884-04

**Authors:** 


### CONTENTS.

ORIGINAL COMMUNICATIONS—

An Address Delivered to the Graduating Class of the Atlanta Medical College. February 28th, 1884. By Wm. H. Felton, M.D., Cartersville, Ga. . . . . . 65
Division of Stricture without Subsequent Sounding. By J. McF. Gaston, M.D., Atlanta, Ga. . . . . . 77
A Pessary Removed from the Vagina after having been worn Eleven Years. By Thos. L. Raines, M.D., Atlanta, Ga. . . . 81
Remarkable Case of Frequent Fractures of Femurs with the Treatment of the Eighth Fracture. By D. H. Howell, M.D., Atlanta, Ga. . . . . . 82
Three Cases of Sympathetic Irido-cyclitis. From Clinic of A. W. Calhoun, M.D. Reported by L. B. V. Woolley, Atlanta, Ga. . . . 84

SELECTIONS—

Case of Congenital Umbilical Hernia Treated by Compression. Recovery. Jordan L. Loyd, F. R. C. S., Eng. . . . . . 86
Experiments with Recently Recommended Remedies in Gonorrhœa. By E. L. Keys, M.D. . . . . . 90
The Relative Merits of Ether and Chloroform as Anesthetics. By J. W. Parkinson, M.D. . . . . . 96
Treatment of Dislocations of the Head of the Humerus. By Hal. C. Wyman, M.D., Detroit . . . . . 102
Infant Feeding and Summer Complaint. By David Little, M.D., Rochester. . . . . . 110

EDITORIAL—

State Medicine . . . . . . 113
Medical Association of Georgia . . . . . . 118
Medical Association of Alabama . . . . . . 119
Tennessee State Medical Society . . . . . . 119
The Sanitarian . . . . . . 119
The Analectic . . . . . . 119
Dr. Lunsford P. Yandell . . . . . . 119
Iodia (Paragraph) . . . . . . 119

NOTES AND COMMENTS—

Observations on Hæmorrhagic Malarial Fever, 120; Poisonous Snakes and their Venom, 122; A Strange Case, 125; Dr. J. Marion Sims, 125; A Case of Monstrosity—a Child with a Dog's Head, 126; Treatment of Earache, 126; A New Method of Administering Quinine to Children, 127; Cotton Candle-Wick Tampons (Foster), 127; The Prognosis in Cases of Post-mortem Wounds, 128.

